# Quantum adiabatic transport in a quantum anomalous Hall insulator

**DOI:** 10.1038/s41467-026-75851-7

**Published:** 2026-07-21

**Authors:** Kajetan M. Fijalkowski, Martin Klement, Nan Liu, Karl Brunner, Charles Gould, Laurens W. Molenkamp

**Affiliations:** 1 Faculty for Physics and Astronomy (EP3), Universität Würzburg, Würzburg, Germany; 2 Institute for Topological Insulators, Würzburg, Germany

**Keywords:** Topological insulators, Quantum Hall

## Abstract

An exceptional trait of the quantum Hall effect is quantum adiabatic transport, dissipationless quantized edge transport that is robust to bias voltages multiple orders of magnitude larger than any known relevant energy scale. This enables stable and highly sensitive measurements in quantum metrology at temperatures above 1 K. In contrast, prior experiments have shown that an electrical bias of the same order applied to the quantum anomalous Hall edge modes in magnetic topological insulators causes a breakdown of quantization, resulting from material limitations (electric field activates bulk transport). In this paper, to mitigate the effects of this electric field and study edge transport at a large electrical bias, we utilize electrochemical potential balancing. We find that electrical transport along the edge of a quantum anomalous Hall insulator is ubiquitously of dissipationless quantum adiabatic nature. In fact, we can verify that the adiabaticity holds at least up to an applied bias voltage of some 600 mV at 4.2 K, multiple orders of magnitude larger than any known energy scale associated with the quantum anomalous Hall state. This is a level of robustness on par with the conventional quantum Hall modes used in mainstream metrology.

## Abstract (web summary)

This work reports on an advancement in achieving robust electrical transport in a topological insulator, which promises applications in metrology, a field of science that defines physical units.

## Introduction

The quantum anomalous Hall effect (QAHE), first observed in the magnetic topological insulator Cr/V-doped (Bi,Sb)_2_Te_3_^1–3^, sparked much research interest in axion electrodynamics^4–8^ and various novel magnetic phenomena^9–15^. In terms of practical applications, the QAHE offers perfectly quantized electrical conductance without the need for an external magnetic field^16–22^, something that has the potential to be a disruptive innovation in the field of quantum metrology as a new type of quantum resistance standard. However, practical implementations remain challenging, since the QAHE is accessible only in a very narrow experimental range of low electrical bias and temperature^2,3,17,23–27^. Nonetheless, previous experiments have shown^23,27,28^ that, while directly quantized Hall resistance is limited to temperatures below some 100 mK, the topological edge states responsible for the QAHE exist up to the Curie temperature (a few tens of K) of the host material, and quantization of Hall resistance is only lost due to the increasing importance of parallel bulk conductance.

One of the remarkable properties of the edge modes in more conventional quantum Hall materials is that they exhibit dissipationless quantum adiabatic transport^29^. An electrical bias of the order of 1 V can be applied to these modes^30,31^ without causing spurious dissipation. Remarkably, this bias, which enables the operation of highly sensitive cryogenic current comparators (CCC) in quantum resistance metrology^30^, is some 4 orders of magnitude larger than the thermal energy scale *k*_B_*T*/*e* at 1 K (*k*_B_ is the Boltzmann’s constant, *e* is the elementary charge and *T* is the temperature) and a few orders of magnitude larger than the typical energy separation between the Landau levels (which is of the order of a few meV for typical quantum Hall measurements^32^). In contrast, when an electrical bias of that order is applied to the topological edges in magnetic topological insulators, a strong non-linear response is observed^28^. A breakdown is observed, presumably due to bulk scattering induced by electric fields in the sample^17,19,21,24–26,33^. Therefore, prior to this work, due to the material limitations, it was not known if the edge transport in a quantum anomalous Hall insulator is also fundamentally of quantum adiabatic nature, or in other words, whether it can sustain as large an electrical bias as conventional quantum Hall modes hosting dissipationless transport. This is the focus of this study.

In order to suppress the effects of strong electric fields, we use a recently developed electrochemical potential balancing scheme using multi-terminal Corbino devices^33^, which allows to eliminate the electric field between the edge modes. This allows us to demonstrate that quantum adiabatic transport in a quantum anomalous Hall insulator is truly ubiquitous, governed by dissipationless electrical transport along the edge, regardless of the magnitude of electrical bias applied across the sample, position of the Fermi level in the band structure, or the sample temperature (as long as the sample is ferromagnetic).

## Results and discussion

**Fig. 1:**
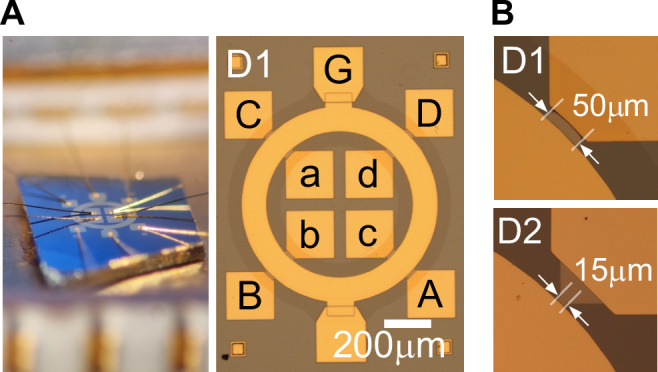
The devices. **A** Left: An angled photograph of device D1. Right: Optical microscope image of the same device (prior to bonding). Letters A–D label the contacts along the outer perimeter, a–d those along the inner perimeter, and G denotes the gate contact. **B** A zoomed-in optical microscope image of the contact geometry for each of the two studied devices D1 and D2.

Our multi-terminal Corbino devices are patterned using standard optical lithography methods, from V_0.1_(Bi_0.2_Sb_0.8_)_1.9_Te_3_ layers grown by molecular beam epitaxy (MBE) on an insulating Si(111) substrate^34^. The Curie temperature of the layers is some 18 K. A device layout is presented in Fig. 1A, along with the labeled contacts. Uppercase letters A-D label the contacts along the outer perimeter, lowercase letters a–d those along the inner perimeter, and G denotes the gate contact. The outer diameter of the Corbino ring is 1 mm, the distance between the inner and the outer perimeters is 100 μm, and the constriction at each electric connection is 50 and 15 μm wide, for devices D1 and D2, respectively. The material composition is optimized for optimal anomalous Hall resistance quantization when properly gated and cooled to temperatures below 100 mK^10,16,21^, as shown in Supplementary Fig. 4A, B.

**Fig. 2:**
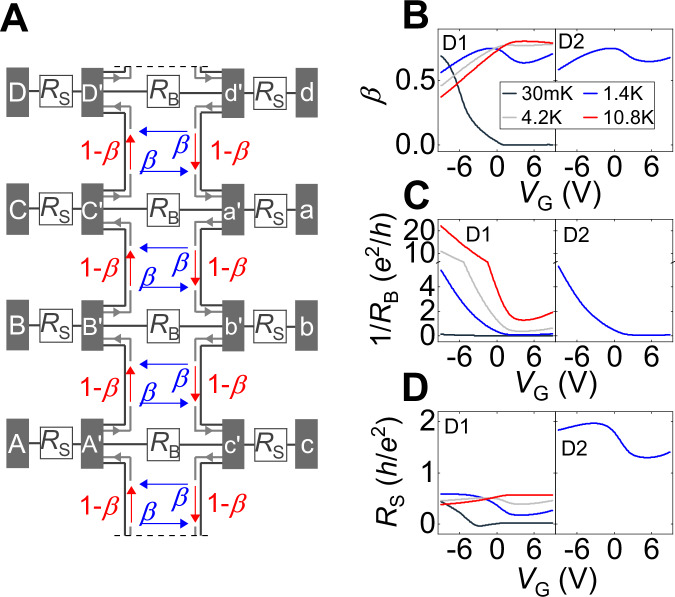
Landauer-Büttiker model. **A** Schematic layout of the Landauer-Büttiker network with three model parameters: inter-edge scattering probability *β*, bulk resistance *R*_B_, and mesa constriction series resistance *R*_S_ (more details in the text and the Supplementary Information). **B**–**D** Gate voltage dependence of the three model parameters for device D1 (left panels) at various temperatures: **B** scattering probability *β*, **C** 1/*R*_B_ (inverse of bulk resistance), and **D** series resistance *R*_S_. In the right panels of (**B**–**D**) are corresponding parameters for device D2 at 1.4 K. The color code is the same for (**B**–**D**).

For modeling, we use a Landauer-Büttiker^35^ formalism-based model adapted for the multi-terminal Corbino geometry^27^. The layout is schematically depicted in Fig. 2A. Electronic transport along the edge is carried by a single dissipationless chiral channel that, in the presence of a finite inter-edge electric field, has a probability *β* to back-scatter via the conducting bulk states to the edge channel located on the opposite edge. We note that the total number of scattered electrons is proportional to the product of *β* and the potential difference between the edges, which is needed to drive a current. Therefore any scattering related to *β* can be fully suppressed, allowing access to adiabatic transport, when the electrochemical potentials of both edges are adjusted to be equal (see later in the text). The parallel bulk conduction is simulated adding resistors *R*_B_ to the circuit (between the nearest neighbor contacts, e.g., A-c), treated in the formalism as additional transmission channels. Given the large aspect ratio of the ring (the circumference is ~28 times larger than the distance between the edges), any bulk path along the ring between next-nearest neighbor contacts (e.g., A-B, A-b, A-d or A-D) has a substantially larger resistance than *R*_B_, and thus is neglected. Finally, in order to account for the finite resistance of the mesa constriction at each electrical connection to the ring, a resistor *R*_S_ is assumed in series with each lead. This series resistance originates from both the edge channel and bulk transport at the constriction. *R*_S_ qualitatively follows the longitudinal resistance of the sample (the longitudinal resistance is shown in Supplementary Fig. 4B). Primed and non-primed indices label the circuit node at each end of this series resistor. Obviously, the potentials at primed and non-primed positions only differ for the source/drain connections, where a current through the constriction creates a voltage drop across it.

**Fig. 3:**
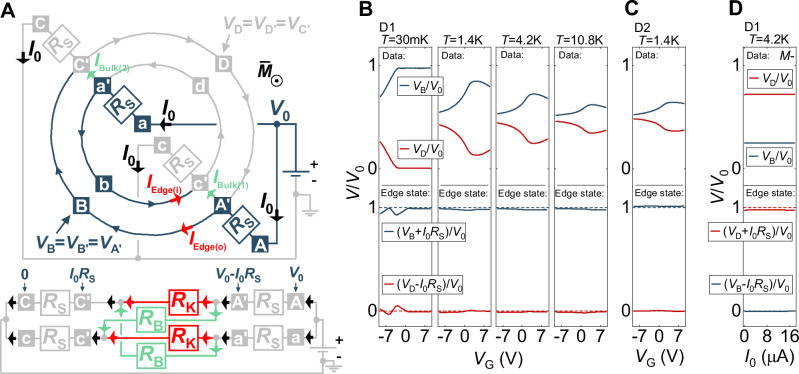
Quantum adiabatic transport. **A** Top: Schematic of the electrical bias circuit. A voltage *V*_0_ is applied to both contacts A and a, whereas contacts C and c are grounded. The dark blue color represents the high electrochemical potential edge of the device, whereas light gray shows the low electrochemical potential, for a positively magnetized state *M*+. Bottom: Equivalent resistor network of the above circuit. *R*_B_ is the bulk resistance parameter, and *R*_K_ represents each chiral edge mode. The colored arrows represent the flowing currents, using the same color code as above. **B** Top: Gate voltage sweep of potentials measured at contacts B (high electrochemical potential) and D (low potential), for device D1, which is biased as in (**A**). The plotted potentials are normalized to the applied bias *V*_0_, which at 30 mK is some 500 μV and above 1 K is some 5 mV. Bottom: The same signals corrected for the voltage drop *I*_0_ *R*_S_ over the mesa constriction through which the current enters/leaves the device. **C** Same as (**B**), for device D2. **D** The same signals as a function of the bias current *I*_0_ for device D1, measured at 4.2 K and a gate voltage of 4 V, and magnetized in the negative direction (*M*−). The colored horizontal dashed lines in (**B**–**D**) represent the values expected for a dissipationless quantum adiabatic transport along the edge. All data is collected at zero external magnetic field.

To determine the parameter values, we compare three measured independent resistance configurations to the formulas calculated from the model. This forms a system of three independent equations with three parameters, which can be solved at each gate voltage and temperature. We outline all details in the Supplementary Information. The gate voltage dependence of each parameter obtained this way is plotted in Fig. 2B–D, for each device D1 and D2. We emphasize that no additional fitting is performed for the analysis in Fig. 3.

Quantum transport effects always come with some characteristic energy scales. Usually, in order to observe a given quantum phenomena, one must ensure that any other energy in the experiment (such as thermal energy or electrical bias) does not exceed the quantization energy scale. In the context of the conventional quantum Hall effect, the relevant energy scale is the separation between the Landau levels (which is of the order of a few meV for typical quantum Hall measurements^32^). Indeed, when the thermal energy (*k*_B_*T*) is large enough to thermally activate carriers into the higher Landau level, the electrical quantization breaks down. Remarkably, this is not the case when the energy comes from the electrical bias. One can apply a voltage of the order of 1 V to the edge modes, which corresponds to energy scale orders of magnitude larger than the Landau level separation, and yet the transport remains perfectly quantized without any spurious dissipation. Actually, this large bias is routinely utilized in high precision quantum Hall experiments, because it enables the stable operation of a CCC, capable of very high resolution measurements^30,31^. This is the quantum adiabatic transport regime, characterized by a full suppression of any inter-edge scattering^29^, protected at the topological level. This enables quantization to persist for a seemingly arbitrarily large electro-chamical potential of the edge.

A natural question is whether the charge transport in a quantum anomalous Hall insulator is similarly robust under large electrical bias, i.e., is it also of quantum adiabatic nature. In previous studies of the QAHE samples, the experimental detection of this kind of transport regime was prohibitive due to material limitations; a breakdown through the bulk states at a relatively low electric field^17,19,21,24–26,33^. Indeed, a measurement of the QAHE breakdown on a Hall bar patterned from nominally identical material as our Corbino devices reveals quantization breakdown already at an electrical bias of a few mV (corresponding to a bias current of some 100 nA, and a current density of some 5 × 10^−4^ A/m)^21^, orders of magnitude lower than that measured using conventional quantum Hall devices based on graphene or GaAs/AlGaAs^30,31^.

To mitigate these material limitations and access the adiabatic regime, we use the recently introduced electrochemical potential balancing method^33^. This is done by applying the bias voltage simultaneously to each perimeter of the device. A constant bias voltage *V*_0_ is applied to both contacts A and a, whereas contacts C and c are grounded, as schematically depicted in Fig. 3A (top). Due to the balance between the applied voltages, there is no difference in electro-chemical potential between the two perimeters around the entire circumference of the device^33^. The potentials at contacts B and b are measured to be equal to within about 1%, and the same is also confirmed for contacts D and d. This in turn suppresses any net electrical current between the edges, since there is no voltage to drive it. This can also be confirmed from the Landauer-Büttiker modeling, where no observable is dependent on the scattering parameter *β* under such a bias setting (see Supplementary Section 3). This constrains the inter-boundary current to flow only directly between contacts A-c and a-C. This ensures that a current *I*_0_ entering the device via contact A on the outer perimeter splits into two independent paths. One path is non-local via the chiral edge channel (*I*_Edge(o)_) sinking to the ground at contact C. The other path is local via the conducting bulk short (*I*_Bulk(1)_) sinking to the ground at contact c. A similar mechanism applies to the current entering the device via contact a on the inner perimeter, which splits into *I*_Edge(i)_ and *I*_Bulk(2)_.

Note that the measured balance between the voltages at the inner and outer perimeters, together with equal total currents entering each perimeter, implies that both edge currents must be equal *I*_Edge(i)_=*I*_Edge(o)_ and both inter-perimeter currents must also be equal *I*_Bulk(1)_=*I*_Bulk(2)_. This is a consequence of the Kirchhoff junction rule of current conservation; see Supplementary Information for details.

If the entire current flowing towards contact B is carried by a dissipationless chiral edge channel sourced from contact A, the electro-chemical potential of contact B is expected to be equal to that at the position ${{\rm{A}}}^{{\prime} }$. This is because the electro-chemical potential is decreased only by a voltage drop over the mesa constriction where the current enters the device. Moreover, the entire edge is expected to be at an equipotential (measurement is shown in Supplementary Fig. 5). Consequently, when a bias voltage *V*_0_ is applied to contact A, the resulting potential at ${{\rm{A}}}^{{\prime} }$, which is then carried on to contact B, is reduced to *V*_0_ − *I*_0_*R*_S_, where *I*_0_*R*_S_ is the voltage drop over the mesa constriction. For the same reason, the potential at ${{\rm{C}}}^{{\prime} }$, which is then carried on to contact D, is accordingly lifted from 0 to *I*_0_*R*_S_. Figure 3B (top) depicts the measured potentials at contacts B and D, collected as a function of the applied gate voltage at various temperatures for device D1. The signals are normalized to the applied bias *V*_0_.

In the bottom panels, we plot the voltages corrected for the expected voltage drop over each source/drain mesa constriction *I*_0_ *R*_S_. *I*_0_ is measured, and *R*_S_ is taken from Fig. 2, without any additional fitting. A voltage *I*_0_ *R*_S_ is added to the measured potential at the high potential contact (B) and subtracted from the measured potential at the low potential contact (D). With this correction, we find that the high potential edge has a potential of 1 (*V*_0_ in absolute units), and the low potential edge a potential of 0, the signals expected for dissipationless transport without any voltage drop along the perimeter. Remarkably, this happens regardless of the value of applied gate voltage or the sample temperature (as long as the material is ferromagnetic).

To further test our analysis, in Fig. 3C, the same procedure as in Fig. 3B is performed for device D2. As a result of having narrower mesa constrictions, and therefore larger *R*_S_, a relatively larger voltage drop over the source and drain constriction is expected. Indeed, in this case the corrected voltages, using *R*_S_ from Fig. 2D, also show a robust value of 1 for the high, and 0 for the low potential edge.

Finally we focus on the robustness of dissipationless edge transport to the magnitude of the applied bias. The bias dependence is plotted in Fig. 3D, measured for device D1 at 4.2 K and a gate voltage of 4 V. For this measurement the layer is magnetized in the negative direction (*M*-), which makes contact D be at higher potential than contact B. The value of *R*_S_ is taken from Fig. 2D at the same temperature and gate voltage, and *I*_0_ is measured. The *I*-*V* characteristic of the device is ohmic within the resolution of our experiment (see Supplementary Fig. 6). In the studied bias range, the *I*-*V* characteristic of the mesa constriction *R*_S_ is linear to within approx. 1% and total voltage applied across the sample is linear to within approx. 0.1%. Similar to the analysis of Fig. 3B, C, *I*_0_ *R*_S_ is added to the voltage measured at the higher potential contact and subtracted from the one at lower potential. It is clear that the same signals characteristic of quantized dissipationless transport remain robust throughout the entire range of the investigated bias, up to *I*_0_ of some 17.5 μA, which corresponds to a total bias voltage *V*_0_ of some 700 mV.

We note that by changing the gate voltage and temperature, the bulk conductance (visible in our analysis as the extracted value of 1/*R*_B_) changes by several orders of magnitude. Despite this fact, the measured potentials ubiquitously agree with the values expected for adiabatic transport. Interestingly, this also shows that the adiabatic transport observed here is rather robust to the effects of Joule heating of the sample caused by the current. The power dissipated reaches as much as 40 μW (*V*_0_ of some 1.3 V and *I*_0_ of some 34 μA at 4.2 K; see below), which is rather substantial for a low temperature quantum system.

Given that the current *I*_0_ splits into the edge current with a conductance of *e*^2^/*h* and inter-perimeter current with a conductance of 1/*R*_B_ = 0.36 *e*^2^/*h* (the value taken from Fig. 2C at this temperature and gate voltage), the resulting edge current represents about 73% of the total current [*I*_Edge _= *I*_0_/(1 + 1/*R*_B_)]. This results in a maximal current injected into the dissipationless edge mode of some 13 μA in this experiment (Fig. 3D). In Supplementary Fig. 3, we also show additional measurements from the same device, collected during another measurement campaign (a separate sample cooldown). There, the maximum total bias voltage applied to the sample is as high as 1.3 V (the voltage limit of the precision amplifiers used to measure potentials). Dissipationless transport without any breakdown is observed in this bias range, which implies that any critical bias value is beyond this maximum value. This results in a current flowing through a quantized dissipationless edge mode *I*_Edge_ as high as 23 μA at 4.2 K, which corresponds to a voltage applied to the edge modes of some 600 mV. This voltage is some 1700 times larger then *k*_B_*T*/*e* at 4.2 K and orders of magnitude larger than any known relevant energy scale associated with the quantum anomalous Hall state. This is a clear evidence for quantum adiabatic transport^29^; dissipationless transport with a full suppression of any inter-edge channel scattering, holding for an almost arbitrarily large electrochemical potential of the edge.

This magnitude of electrical current is already comparable to that routinely used in Hall bars exhibiting conventional quantum Hall effect in metrology^30,31^. Given how much of an improvement electrochemical potential balancing brings for the edge transport in a quantum anomalous Hall insulator, it will be interesting to see the influence this method will have on the robustness of the conventional quantum Hall effect. Once this method is implemented there, it will also be informative to quantitatively compare the robustness of both effects without the negative effects of inter-perimeter electric field.

We have studied non-local signals on multi-terminal Corbino devices based on V-doped (Bi,Sb)_2_Te_3_ under an electrochemical potential balancing scheme. We find that electrical transport in a quantum anomalous Hall insulator is ubiquitously of quantum adiabatic nature, regardless of sample temperature, position of the Fermi level, or the magnitude of electrical bias, tested up to a voltage of some 600 mV at 4.2 K. This not only demonstrates the ultimate robustness of topologically protected transport, but also places the capabilities of the quantum anomalous Hall edge modes in our material on par with the conventional integer quantum Hall modes used in mainstream metrology^30,31^. Our results thus show that a primary quantum standard of resistance operating at 4.2 K and zero external magnetic field based on the QAHE is possible, provided the bulk of the magnetic topological insulator can be made sufficiently insulating at that temperature.

## Supplementary information

Supplementary Information

Transparent Peer Review File

## Source data

Source Data

## Data Availability

All data necessary to support the conclusions of the paper are available in the manuscript. Source data are provided with this paper.
