# Supplementary material for: Quantum adiabatic transport in a quantum anomalous Hall insulator
Source: Nat Commun. 2026 Jul 21;17:6827. doi: 10.1038/s41467-026-75851-7 (PMC13388678; doi:10.1038/s41467-026-75851-7)
Supplement: Supplementary file 1 — Supplementary Information [file 41467_2026_75851_MOESM1_ESM.pdf]

Quantum adiabatic transport in a quantum anomalous Hall insulator - Supplementary Information

Kajetan M. Fijalkowski, Martin Klement, Nan Liu, Karl Brunner, Charles Gould and Laurens W. Molenkamp
[1] *Faculty for Physics and Astronomy (EP3), Universität Würzburg, Am Hubland, D-97074, Würzburg, Germany*
[2] *Institute for Topological Insulators, Am Hubland, D-97074, Würzburg, Germany*

TABLE OF CONTENTS:

1. The Landauer-Büttiker formalism-based model.
2. Determining the parameter values.
3. Solution for a balanced bias on each perimeter.
4. On both bulk currents being equal and both edge currents being equal.
5. Non-local measurements at even larger bias current (on a separate cooling of the sample).
6. Basic characterization of the devices.
7. Measurement of the equipotential along the edge.
8. *I*-*V* characteristics of the device.

Supplementary Section 1 THE LANDAUER-BÜTTIKER FORMALISM-BASED MODEL

The Landauer-Büttiker network schematically depicted in Fig. 2A of the main text (for ease of navigation also in the Supplementary Fig. 1) can be expressed as a system of 8 linear equations relating the flowing currents with the lead potentials for a positively magnetized state ($M+$):

$$\begin{bmatrix} I_\mathrm{A} \\ I_\mathrm{B} \\ I_\mathrm{C} \\ I_\mathrm{D} \\ I_\mathrm{c} \\ I_\mathrm{b} \\ I_\mathrm{a} \\ I_\mathrm{d} \end{bmatrix} = \frac{e^2}{h} \begin{bmatrix} T_1 & T_2 & 0 & 0 & T_3 & 0 & 0 & 0 \\ 0 & T_1 & T_2 & 0 & 0 & T_3 & 0 & 0 \\ 0 & 0 & T_1 & T_2 & 0 & 0 & T_3 & 0 \\ T_2 & 0 & 0 & T_1 & 0 & 0 & 0 & T_3 \\ T_3 & 0 & 0 & 0 & T_1 & 0 & 0 & T_2 \\ 0 & T_3 & 0 & 0 & T_2 & T_1 & 0 & 0 \\ 0 & 0 & T_3 & 0 & 0 & T_2 & T_1 & 0 \\ 0 & 0 & 0 & T_3 & 0 & 0 & T_2 & T_1 \end{bmatrix} \begin{bmatrix} V_\mathrm{A} \\ V_\mathrm{B} \\ V_\mathrm{C} \\ V_\mathrm{D} \\ V_\mathrm{c} \\ V_\mathrm{b} \\ V_\mathrm{a} \\ V_\mathrm{d} \end{bmatrix} \quad (1)$$

and for a negatively magnetized state (M-):

$$\begin{bmatrix} I_A \\ I_B \\ I_C \\ I_D \\ I_c \\ I_b \\ I_a \\ I_d \end{bmatrix} = \frac{e^2}{h} \begin{bmatrix} T_1 & 0 & 0 & T_2 & T_3 & 0 & 0 & 0 \\ T_2 & T_1 & 0 & 0 & 0 & T_3 & 0 & 0 \\ 0 & T_2 & T_1 & 0 & 0 & 0 & T_3 & 0 \\ 0 & 0 & T_2 & T_1 & 0 & 0 & 0 & T_3 \\ T_3 & 0 & 0 & 0 & T_1 & T_2 & 0 & 0 \\ 0 & T_3 & 0 & 0 & 0 & T_1 & T_2 & 0 \\ 0 & 0 & T_3 & 0 & 0 & 0 & T_1 & T_2 \\ 0 & 0 & 0 & T_3 & T_2 & 0 & 0 & T_1 \end{bmatrix} \begin{bmatrix} V_\mathrm{A} \\ V_\mathrm{B} \\ V_\mathrm{C} \\ V_\mathrm{D} \\ V_\mathrm{c} \\ V_\mathrm{b} \\ V_\mathrm{a} \\ V_\mathrm{d} \end{bmatrix} \quad (2)$$

where T_1=1+1/R_B (R_B is resistance in units of h/e^2), T_2=-(1-β), and T_3=-(β+1/R_B). The influence of bulk conductance is simulated using two parameters: R_B and β. In order to account for a series resistance R_S from the mesa constriction, the potential at each lead (except for the source and drain leads) needs to be adjusted by a voltage drop over the drain mesa constriction IR_S, where I is a bias current flowing through the drain contact to the ground.

Supplementary Section 2 DETERMINING THE PARAMETER VALUES

The numerical value for each of the three parameters (β, R_B, R_S) can be determined by equating the three experimentally measured resistances ($R_{\mathrm{A-c,A-c}}$=$V_{\mathrm{A-c}}/I_{\mathrm{A-c}}$, $R_{\mathrm{A-C,A-C}}$=$V_{\mathrm{A-C}}/I_{\mathrm{A-C}}$, $R_{\mathrm{B-D,A-C}}$=$V_{\mathrm{B-D}}/I_{\mathrm{A-C}}$) with their calculated forms. Each resistance is measured using a low bias d.c. voltage excitation (a few mV at 1.4 K and above, a few hundreds of μV at 30 mK). The solution of the Landauer-Büttiker network for each of the three resistance configurations is:

$$R_{\mathrm{A-c,A-c}} = \frac{h}{e^2}\frac{3\beta+1+4/R_\mathrm{B}}{4(\beta+1/R_\mathrm{B})(1+1/R_\mathrm{B})} + 2R_\mathrm{S} \quad (3)$$

$$R_{\mathrm{A-C,A-C}} = \frac{h}{e^2}\frac{1}{1-\beta} + 2R_\mathrm{S} \quad (4)$$

$$R_{\mathrm{B-D,A-C}} = \frac{h}{e^2}\frac{1}{1+1/R_{\mathrm{B}}} \tag{5}$$

which form a system of three independent equations. Substituting the left hand side of each equation with an experimentally measured resistance (Supplementary Fig. 2A) determines the value for each parameter (Supplementary Fig. 2B). We emphasize that the three configurations are distinct from the ones analyzed in the main text Fig. 3 and no additional fitting is performed for the analysis in Fig. 3.

The series resistance of the mesa constriction R_{S} qualitatively follows the longitudinal resistance (longitudinal resistance is plotted in Supplementary Fig. 4B). This is because the resistance of a mesa constriction at each contact is a result of parallel edge channel and bulk conductance.

The slightly negative value of R_{S} right at the edge of the QAHE plateau at 30 mK (Supplementary Fig. 2B) is most likely an artifact resulting from non-uniformity of the bulk resistivity along the ring when the system is barely at the mobility edge. Our model assumes a perfectly symmetric device and homogeneous material, and spatial variations in material properties will introduce uncertainties. Importantly, the negative R_{S} does not occur at higher temperatures where inhomogeneities are effectively erased out by the conducting bulk and charge transport is uniform, as clearly shown by the quality of the data and the model analysis in Fig. 3 in the main text. This observation implies that the model gives an accurate description of transport in that regime.

The bulk resistance R_{B} has a clear maximum around 4 V, which coincides with the gate voltage range for which optimal QAHE is observed at lower temperatures. The inter-edge scattering probability β decreases monotonically with increasing the bulk resistance R_{B} (or equivalently β is monotonic with bulk conductance $1/R_{\mathrm{B}}$) around a gate voltage of 4 V, where the bulk is most insulating. The drop in value of β observed for large negative gate voltages at higher temperatures is likely a consequence of additional parallel bulk channels connecting the scattered electrons directly into various leads across the device, unaccounted for in this simple model. We note that any scattering related to β is suppressed when electrochemical potential balancing is used (see section 3 of this Supplementary Information).

Since the only difference in geometry between the two devices is the mesa constriction width, when measurements are taken under nominally the same conditions, one expects the parameters β and R_{B} to remain unchanged, and only the series resistance R_{S} to vary. Indeed this is reflected in Supplementary Fig. 2B, where the blue curves correspond to 1.4 K for each device. In addition, R_{S} is considerably larger for a device D2, as expected due to narrower constrictions.

Supplementary Section 3
SOLUTION FOR A BALANCED BIAS ON EACH PERIMETER

For a bias voltage V_0 applied to contacts A and a (and current I_0 flowing into each), and contacts C and c grounded, one obtains the following voltages along the device for the positively magnetized state (M+):

$$V_{\mathrm{A}} = V_{\mathrm{a}} = V_0 = I_0\frac{h}{e^2}\frac{1}{1+1/R_{\mathrm{B}}} + 2I_0R_{\mathrm{S}} \tag{6}$$

$$V_{\mathrm{B}} = V_{\mathrm{b}} = I_0\frac{h}{e^2}\frac{1}{1+1/R_{\mathrm{B}}} + I_0R_{\mathrm{S}} \tag{7}$$

$$V_{\mathrm{C}} = V_{\mathrm{c}} = 0 \tag{8}$$

$$V_{\mathrm{D}} = V_{\mathrm{d}} = I_0R_{\mathrm{S}} \tag{9}$$

Note that as expected the result is independent of the scattering parameter β. In addition V_{B}=V_0-I_0R_{S} and V_{D}=I_0R_{S}, which is central to the analysis in Fig. 3 of the main text.

Supplementary Section 4
ON BOTH BULK CURRENTS BEING EQUAL AND BOTH EDGE CURRENTS BEING EQUAL

For a double-biased scenario with two sources and two grounded leads, the current splits (or merges) at four points on the circuit. From the source contacts, two equations follow from the current conservation. For contact A:

$$I_0 = I_{\mathrm{Edge(o)}} + I_{\mathrm{Bulk(1)}} \tag{10}$$

and for contact a:

$$I_0 = I_{\mathrm{Edge(i)}} + I_{\mathrm{Bulk(2)}} \tag{11}$$

From the grounded contacts, two equations follow. For contact C:

$$I_{\mathrm{Edge(o)}} + I_{\mathrm{Bulk(2)}} = I_0 \tag{12}$$

and for contact c:

$$I_{\mathrm{Edge(i)}} + I_{\mathrm{Bulk(1)}} = I_0 \tag{13}$$

It trivially follows from equations (10-13) that $I_{\mathrm{Edge(i)}}$=$I_{\mathrm{Edge(o)}}$ and $I_{\mathrm{Bulk(1)}}$=$I_{\mathrm{Bulk(2)}}$.

Supplementary Section 5
NON-LOCAL MEASUREMENTS AT EVEN LARGER BIAS CURRENT (ON A SEPARATE COOLING OF THE SAMPLE)

Supplementary Fig. 3 shows the non-local measurements tested up to larger sample bias I_0, for device D1 at a temperature of 4.2 K and 10 K. The data was collected during different cooldown (separate from the data discussed in the main text and elsewhere in the supplement). In order to account for minor cooldown-to-cooldown variations in the sample properties, the model parameters for this cooldown are separately calibrated following the same procedure as discussed in section 2. The resulting parameter values (series resistance R_S and bulk resistance R_B) for each plotted gate voltage and temperature are listed in the Figure. In this experiment, the maximum current injected into the quantized edge state is about 23 μA at 4.2 K (with the total current I_0 of some 34 μA and total voltage applied over the sample V_0 of some 1.3 V) and 13 μA at 10 K (both at a gate voltage of 4V).

Supplementary Section 6
BASIC CHARACTERIZATION OF THE DEVICES

Supplementary Fig. 4 shows the results of basic characterization (Hall and longitudinal resistance) of the devices D1 and D2. When the samples are cooled to some 30 mK, the Hall resistance is quantized to $\pm h/e^2$ and longitudinal resistance drops to 0, as expected for the quantum anomalous Hall effect.

Supplementary Section 7
MEASUREMENT OF THE EQUIPOTENTIAL ALONG THE EDGE

Supplementary Fig. 5 shows measurements of an equipotential along the edge (the same potential measured at adjacent contacts B and C), under balanced voltage bias.

Supplementary Section 8
I-*V* CHARACTERISTICS OF THE DEVICE

Supplementary Fig. 6 shows the measured potential difference between contacts A and B (V_A-V_B), as well as a total voltage drop across the sample (V_A), plotted as a function of the bias current. The characteristic V_A-V_B is linear to within approx. 1 %, and the total voltage V_A is linear to within approx. 0.1 %. Since contact B probes the potential at node A' (due to the dissipationless chiral edge channel connecting and equilibriating B and A'), the voltage drop V_A-V_B gives a direct measure of the voltage drop over the mesa constriction R_S (as well as a possible series contact resistance).

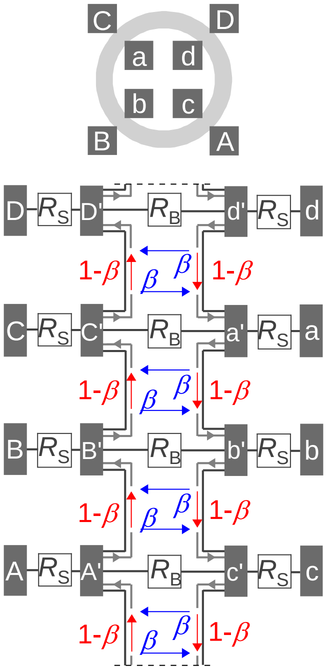

Supplementary Fig. 1. **Landauer-Büttiker network.** Schematic layout of the Landauer-Büttiker network with three model parameters: inter-edge scattering probability β, bulk resistance R_{B}, and series resistance of the mesa constriction R_{S}.

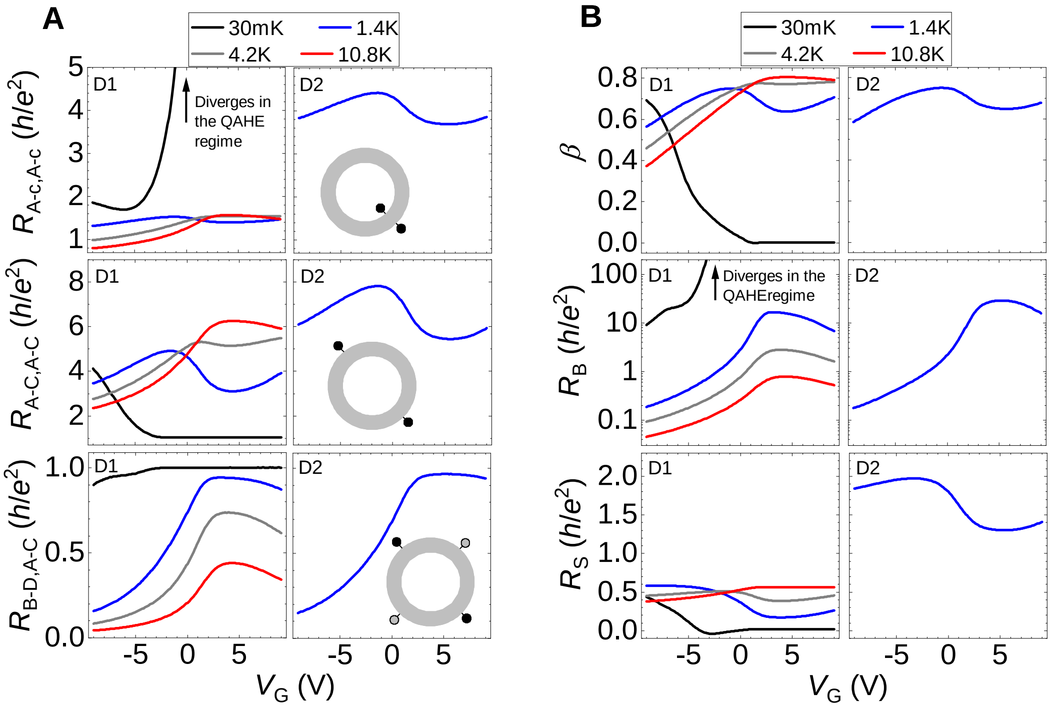

Supplementary Fig. 2. **Measured resistance values used to extract the parameters.** A) The three resistance configurations for each device, D1 and D2, measured at varous temperatures and zero external magnetic field. B) The corresponding extracted values of each parameter.

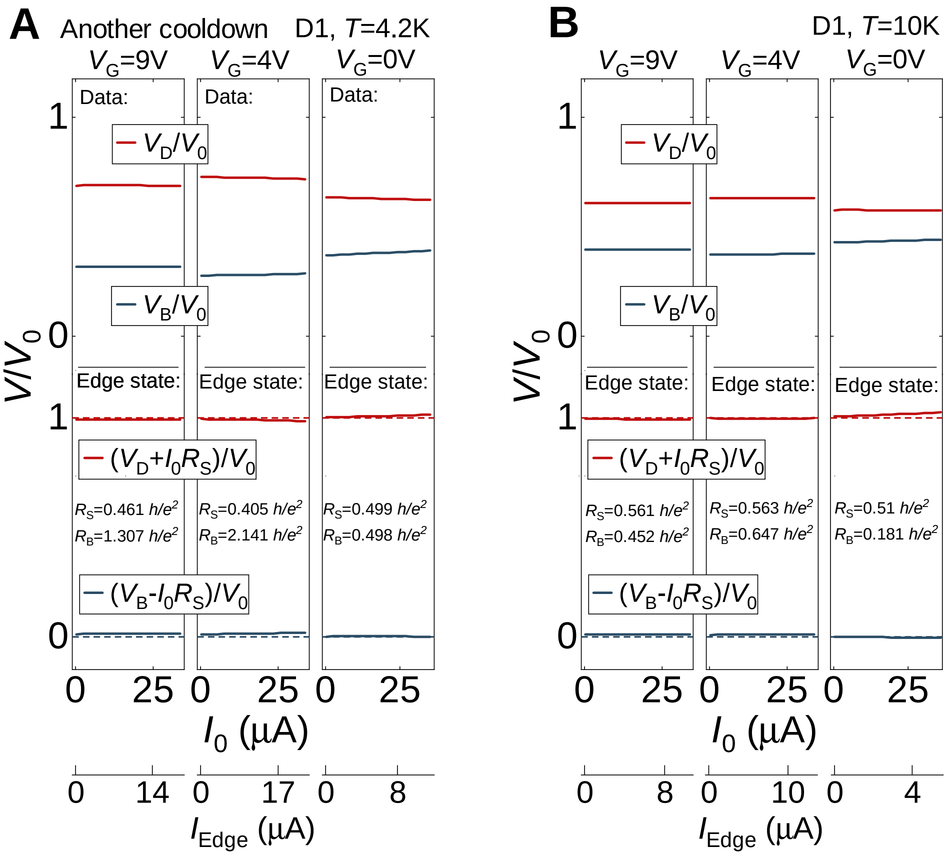

Supplementary Fig. 3. **Testing the quantum adiabatic transport at a larger electrical bias.** A) Top: Bias current I_0 dependence of the potentials measured at contacts B (low electrochemical potential) and D (high potential), collected from the device D1 at 4.2 K and various gate voltages. The plotted potentials are normalized to the applied bias voltage V_0. Bottom: The same signals corrected for the voltage drop $I_0 R_S$ over the source and drain mesa constrictions. B) The same at 10 K. The data was collected at zero external magnetic field, for the state magnetized in the negative direction (M-). Colored horizontal dashed lines represent the values expected for a dissipationless QAHE chiral edge transport. The additional horizontal axis mark current that flows through the edge state $I_{\mathrm{Edge}}=I_0/(1+1/R_B)$ for each gate voltage and temperature. The data was collected during another cooldown of the sample (separate from the measurements plotted in other figures).

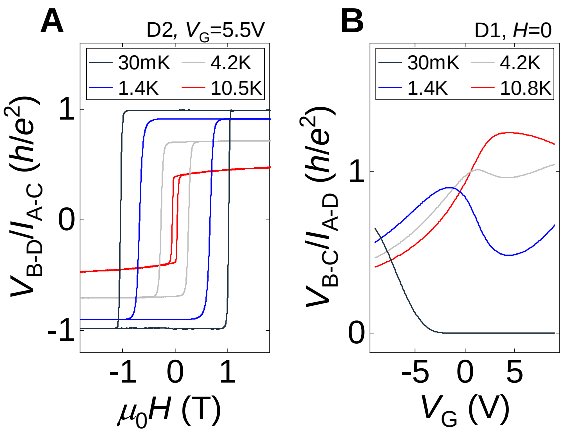

Supplementary Fig. 4. **Basic characterization of the devices.** A) Hall resistance ($V_{\mathrm{B-D}}/I_{\mathrm{A-C}}$) from device D2 as a function of an external magnetic field applied perpendicular to plane, collected at various temperatures and applied gate voltage of 5.5 V. B) Gate voltage sweep of the longitudinal resistance ($V_{\mathrm{B-C}}/I_{\mathrm{A-D}}$) of device D1, collected at various temperatures and zero external magnetic field.

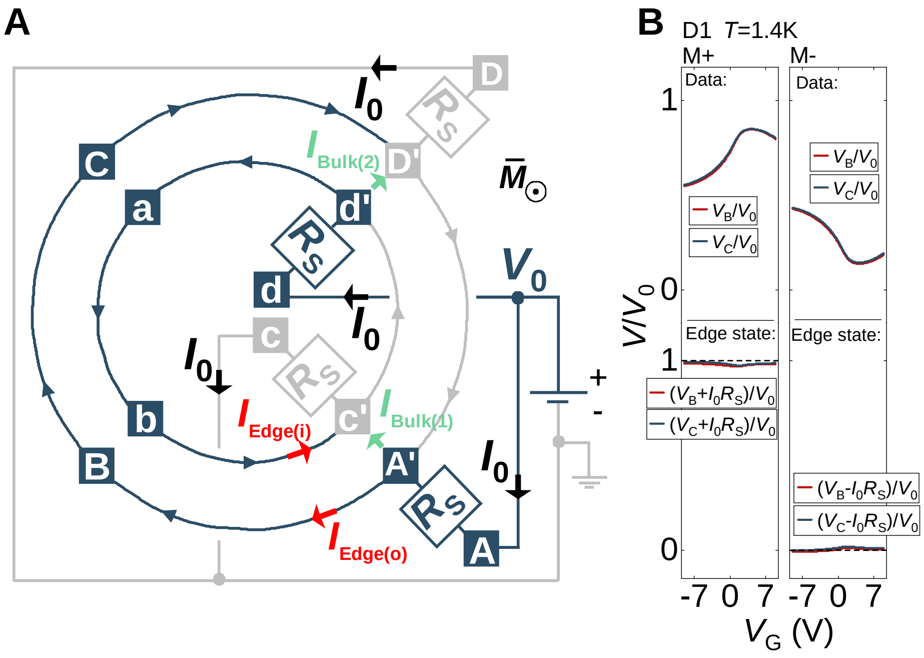

Supplementary Fig. 5. **Equipotential along the perimeter.** A) Schematic of the electrical bias circuit. A voltage V_0 is applied to both contacts A and d, whereas contacts c and D are grounded. B) Top: Gate voltage sweep of potentials measured at contacts B and C, for device D1 at 1.4 K, which is biased as in (A), for a sample magnetized along each perpendicular to plane direction. The plotted potentials are normalized to the applied bias V_0. Bottom: The same signals corrected for the voltage drop $I_0 R_S$ over the source/drain mesa constrictions. The horizontal dashed lines in (B) represent the values expected for a dissipationless quantum adiabatic transport along the edge. Data is collected at zero external magnetic field.

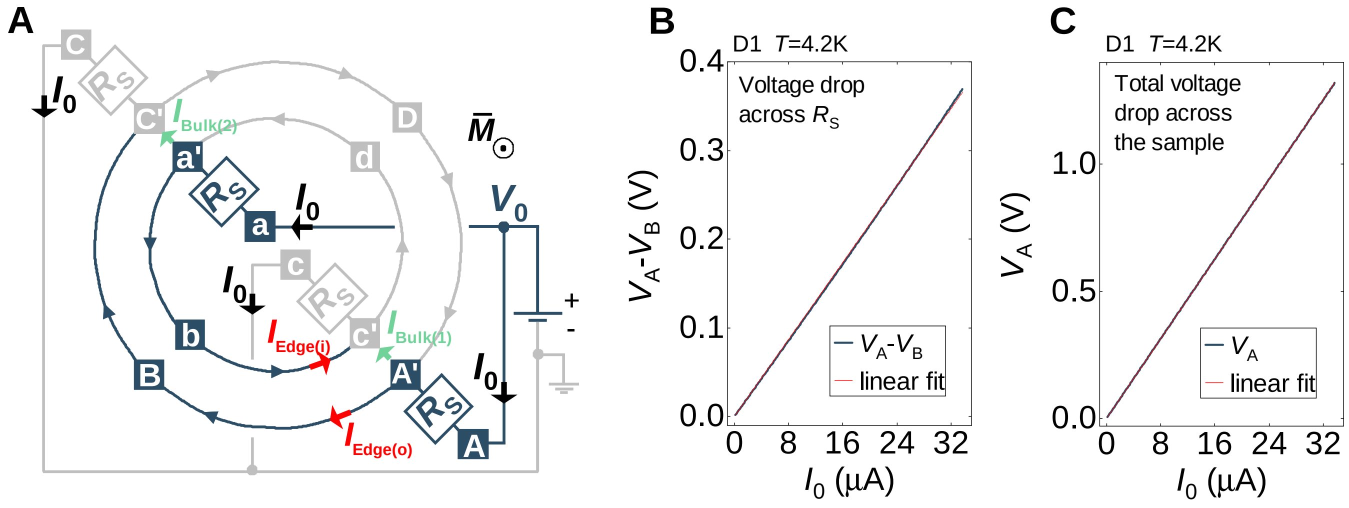

Supplementary Fig. 6. *I*-*V* **characteristics of the device under the electrochemical potential balancing bias scheme.** A) Schematic of the electrical bias circuit. A voltage V_0 is applied to both contacts A and a, whereas contacts c and C are grounded. B) Measured potential difference V_A-V_B (blue line), plotted as a function of the bias current I_0, together with a linear fit (red line). Data is collected from device D1 at 4.2 K, a gate voltage of 4 V, and zero external magnetic field. C) The same for a total voltage across the sample V_A.
