# Supplementary material for: Quantum adiabatic transport in a quantum anomalous Hall insulator
Source: Nat Commun. 2026 Jul 21;17:6827. doi: 10.1038/s41467-026-75851-7 (PMC13388678; doi:10.1038/s41467-026-75851-7)
Supplement: Supplementary file 2 — Transparent Peer Review File [file 41467_2026_75851_MOESM2_ESM.pdf]

nature portfolio

Peer Review File

Quantum adiabatic transport in a quantum anomalous Hall insulator

Corresponding Author: Dr Kajetan Fijalkowski

This file contains all reviewer reports in order by version, followed by all author rebuttals in order by version.

Version 0:

Reviewer comments:

Reviewer #1

(Remarks to the Author)
What are the noteworthy results?

The quantum anomalous Hall effect (QAHE), discovered in recent years, has attracted significant attention because of its potential applications in metrology, such as resistance standards under zero magnetic field. However, compared to the integer quantum Hall effect, practical implementation is still challenging due to a relatively small critical current, leading to skepticism regarding its feasibility.
In this paper, the authors fabricated multi-terminal Corbino devices from V-doped BiSbTe exhibiting the QAHE. By eliminating the electric field between the edge modes using electrochemical potential balancing developed by the authors, the robustness of the QAHE edge channels was investigated. The most noteworthy result is that quantum edge modes arising from the QAHE were observed even under large current bias, revealing that the edge modes are significantly more stable than previously expected. This result provides a hopeful outlook for further optimization of the QAHE in future applications.

Will the work be of significance to the field and related fields? How does it compare to the established literature? If the work is not original, please provide relevant references.

The result observed in this study is particularly significant: the quantum anomalous Hall effect (QAHE) was maintained even under current levels of several tens of microamperes, which is significantly higher than previously expected.
This demonstrates that the QAHE possesses a robustness comparable to that of the integer quantum Hall effect. Improving the quality of QAHE devices has been a global challenge, and skepticism regarding its practicality still remains.
The results findings provide a promising outlook for enhancing the stability of the QAHE in future experiments.

Does the work support the conclusions and claims, or is additional evidence needed?
Yes.
The material, device fabrication, and low-temperature transport measurements presented in this study are all of a very high standard, and the experimental results presented are sufficient to support the conclusions and claims.

Are there any flaws in the data analysis, interpretation and conclusions? Do these prohibit publication or require revision?
No.

Is the methodology sound? Does the work meet the expected standards in your field?
Yes.

Is there enough detail provided in the methods for the work to be reproduced?

Comment1: How did the authors determine the values shown in Figs. 2B–2D? Were they calculated at a specified current, or obtained by fitting the I–V curves presented in Supplementary Information Fig. 2A?

Comment2: I recommend that the authors provide information regarding the quality of the material. In the case of two-dimensional electron gases (2DEGs) such as GaAs/AlGaAs or graphene, the quality is typically characterized by carrier density and mobility. Is it possible to specify the material quality in a similar manner? Alternatively, could the authors estimate the critical current density for the breakdown of the QAHE if a usual Hall bar (rather than a Corbino geometry) were fabricated from the same material?

Reviewer #2

(Remarks to the Author)
The main claim of the paper is that the electrical transport in a quantum anomalous Hall insulator (QAHI) device, which employs the electrochemical potential balancing between the chiral edge modes, is "ubiquitously of quantum adiabatic nature, regardless of the temperature, position of the Fermi level in the band structure, or the electrical bias across the sample". The practical consequence of this approach, according to authors, could be a possibility of highly sensitive measurements in quantum metrology at temperatures above 1 K. I do not find the authors' analysis of the presented data and the conclusions drawn from it sufficiently convincing and do not recommend publishung their work in Nature Communications. Below I present my arguments:

1) Concerning the electrochemical potential balancing between the chiral edge modes, this is indeed interesting approach, which allows to avoid building up a significant electrical field between counter-propagating edge modes along the inner and outer perimeters of the Corbino device. However, it is still impossible to avoid the separation of the high and low electrochemical potentials at contacts where the current enters and exits the device. In this study, this separation at contacts A, a, C, and c is 50 mkm in the device 1 and 15 mkm in the device 2. One would expect the breakdown of QAHI state at these contacts when the current accedes a critical value. This is not fundamentally different from the situation in conventional Hall-bar devices.

2) Concerning the Landauer-Büttiker description of the mixed transport through the edge modes and the bulk channels, authors introduce a model with three parameters: \beta is a probability of scattering from an edge mode to a contra-propagating edge mode on the other perimeter of the Corbino device; R_B is an effective bulk resistance, accounting for N=1/R_B bulk channels; R_c is a contact resistance, which is assumed to be the same for all contacts. Authors treat these parameters as independent ones and obtain them from the three measured resistances in configurations shown in Supplementary Fig. 2 (three parameters from the system of three equations).
There are several problems with this model and this approach:
(i) Parameters are not independent. Both \beta and R_B related to the bulk conduction and cannot be independent from each other. This can be seen, e.g., from the equation (4) in Supplementary Materials, which essentially gives the resistance of a two-probe measurement of a QAHI sample with a hole. If R_B is finite, \beta cannot be zero, otherwise the resistance will be quantized (h/e^2 + 2R_C), even though the sample has bulk conducting channels.
(ii) It is not clear why bulk channels are connected to only opposite contacts on the inner- and outer-perimeters. As one can read from the Landauer-Büttiker conduction matrices [eqs. (1) and (2) in Supplementary Material], all N=1/R_B bulk channels, which goes into a particular contact, bring only the electrochemical potential of the opposite contact.
(iii) Contact resistance R_C seems to be one of the most important parameters in the proposed approach. It allows by simply adding I_0*R_C to V_B and subtracting I_0*R_C from V_D to obtain effective voltages, which are independent from the gate voltage, the temperature or the bias current and their difference gives the quantized Hall resistance (as shown in the Fig. 3). Judging from the gate and temperature dependencies of R_C, it does not look as a real contact resistance, but rather as some effective parameter without clear physical meaning. R_C can be even negative at gate voltage of -2.7V as one can see in Supplementary Fig. 2 B. On the other hand, real contact resistances of this device can be easily measured in the three-probe configuration, at least at 30 mK, when QAHI state is not broken.

3) In general, the proposed approach is as follows: from resistances measured in several configurations on a Corbino device, certain parameters (whatever their physical meaning) are extracted and then all transport components, including the contribution of edge modes, are reconstructed.
The accuracy of the reconstruction depends on the model and sample details, making this approach fundamentally problematic for quantum metrology, which the authors invoke as a motivation for their study.
The claim of the quantum adiabatic nature of the transport in presented device is also not justified, in my opinion. My understanding is that the adiabaticity in QHE (QAHI) systems implies dissipationless 1D edge transport which is responsible for a precise quantization of the Hall resistance. The gate voltage, the temperature or the current can break this state, and I do not see how the proposed reconstruction can change this fact. Authors need to make it clear what they mean by the adiabaticity, at least make a section in Supplementary Material.

4) What is the point of comparing the bias voltage with the thermal energy scale k_B*T/e?

Version 1:

Reviewer comments:

Reviewer #1

(Remarks to the Author)

The authors have responded to my comments thoroughly. The manuscript is of very high scientific quality. I can now support publication.

Reviewer #2

(Remarks to the Author)
The authors have made significant revisions to the manuscript and addressed most of my comments, but some questions remain. In their response, authors emphasize that the paper is not aimed at building a metrological standard, but rather focus on the robustness of the QAH edge states, for which authors added the explanation of the idea of quantum adiabaticity in several parts of the main text and SM.

Nevertheless, the metrology aspect remains the primary motivation for paper, and the question remains as to how accurate the quantization of the Hall resistance is at different temperatures, gate voltages, and applied bias currents. Authors claim that “any possible breakdown at the mesa constriction (where the current enters/leaves the device) merely introduces an additional series resistance that is eliminated in a four-terminal measurement geometry. When that happens, the Hall resistance remains quantized”. I doubt this statement. It is based on the reconstruction of the edge and the bulk contributions to the conductance from three experimentally measured resistances R_A–c,A–c, R_A–C,A–C, and R_B–D,A–C as shown in Fig.3. The estimation of the edge contribution close to e^2/h is not a surprise, simply because it was implemented into the Landauer-Büttiker model.

This reconstruction also plays a central role in the consideration of the transport adiabaticity. In particular, authors added the following statement in the revised version of the manuscript: the applied “voltage is some 1700 times larger then kBT/e at 4.2 K and orders of magnitude larger than any known relevant energy scale associated with the quantum anomalous Hall state. This is a clear evidence for quantum adiabatic transport [29]; dissipationless transport with a full suppression of any inter-edge channel scattering, holding for an almost arbitrarily large electrochemical potential of the edge.”
The concept of adiabatic transport in the field of QHE, which was reviewed in the seminal paper by Beenakker and van Houten and cited in the paper as Ref. [29], is related to the suppression of the inter-edge channel scattering, which can destroy the quantization of the transport response (quantized Hall resistance and zero longitudinal one). The edge channel in QAHI is obviously differs from the edge channels in QHE (with possible exception of the state with a filling factor one). Nevertheless, the scattering between counter-propagating edge modes on the inner- and outer-perimeters of the Corbino device can destroy the adiabatic nature of the edge modes. In fact, the reconstruction of transport parameters (Fig.2) shows finite values of the parameter \beta (except for the lowest temperature 30 mK and the Fermi level position within the magnetic gap). This is a direct indication of a finite scattering probability between these two edge modes, pointing on the possible loss of the adiabatic edge transport.

Another point that requires further consideration/clarification in the paper concerns the assumption that the applied bias voltage multiplied by the elementary charge represents the relevant energy scale for adiabatic quantum transport. Even in the quantum Hall effect, such an energy scale cannot be directly applied for comparison with the Landau level separation energy. Why should it be relevant for the quantum anomalous Hall effect? In the quantum point contact example given by authors in in their response, temperature and applied bias voltage are also not the only relevant parameters. Arguably the spatial dependence of the confinement potential plays a more important role. So, the relevance of the energy scale in the nature of the adiabatic transport should be properly justified.

Despite the questions raised, the overall conclusion of the paper—that the edge modes in the ferromagnetically ordered QAHI state are quite robust—is plausible. After all, the typical temperature dependences of the resistivities in usual QAHI Hall-bar samples show a rapid increase of the anomalous Hall signal in R_yx and a rapid decrease of R_xx, indicating an increasing contribution to the conductance from the edge channel which is at least comparable with the bulk contribution even at temperatures close to the Curie temperature. And this behavior does not depend much on the current, which can be easily tens of microamps.

Overall, this work is a fairly valuable contribution to the QAHE research, however, in my opinion, the reported results are not important enough to justify publication in Nature Communication.

Version 2:

Reviewer comments:

Reviewer #2

(Remarks to the Author)
The authors' response didn't change my opinion of the manuscript, and their sarcasm —" The Referee apparently does not understand the basics of a four-terminal measurement … known since 1861, when Lord Kelvin invented the four-terminal measurement technique "— isn't helping either. While I don't support the publication of this article in Nature Communications, I think it's only fair that readers form their own opinions about the scientific merit of this work.

Reviewer #3

(Remarks to the Author)
This manuscript wirtten by K. M. Fijalkowski et al. reports the observation of quantum adiabatic transport in a quantum anomalous Hall insulator using a multi-terminal Corbino geometry combined with an electrochemical potential balancing scheme. The authors demonstrate that dissipationless edge transport persists under electrical bias up to ~600 mV at 4.2 K, substantially exceeding previously reported bias limits in QAHE systems. The work is technically impressive and of potential importance for quantum metrology and the understanding of topological edge transport. The manuscript is well written, and the experimental methodology appears sound. I recommend its publication in Nature Communication, provided the authors address the following comments.
1.At a maximum bias voltage of 600 mV and a current of around 17.5uA, the total injected power into the mesoscopic device exceeds 10uW. While the edge modes themselves are dissipationless, the current flowing through the bulk and the series mesa constrictions will inevitably produce Joule heating. Does this localized heating affect the bulk resistivity or the inter-edge scattering probability? A brief discussion on the thermal dissipation dynamics would solidify the robustness claim.
2.The conclusion of quantum adiabatic transport appears to rely on the agreement between the measured contact potentials and the Landauer-Büttiker model after correcting for the series resistance Rs. Could the authors comment on how sensitive is the conclusion to uncertainties in the extracted Rs values? Does Rs remain Ohmic up to 17.5 uA? Non-linearities in the contact and constriction resistance at high bias could undermine the accuracy of the V\pm I0Rs correction, It would be helpful if the authors could provide an I-V curve of the constriction itself or demonstrate the constancy of Rs at higher bias current.
3.Although the electrochemical potential balancing scheme suppresses the net inter-edge current, a finite bulk conductance RB-1 is still present in the model. Could the authors further clarify to what extent residual bulk transport may influence the extracted edge potentials, particularly at elevated temperatures or large bias?
4.The authors state that dissipationless transport holds “at least up to an applied bias voltage of ~600 mV” (or ~700 mV for I0 = 17.5 uA). Did the authors directly observe a breakdown of quantization beyond this voltage, or is this limit set by more trivial experimental constraints?
5.The comparison with conventional quantum Hall systems is interesting. Could the authors provide a more quantitative comparison in terms of current density, breakdown thresholds, etc, to better establish the claimed equivalence in metrological performance?

Version 3:

Reviewer comments:

Reviewer #3

(Remarks to the Author)
The authors have addressed all of my questions. I recommend this manuscript for publication in Nature Communications.

Reviewer #4

(Remarks to the Author)
Previous experiments [for example, PRB 106, 045419 (2022)] found a current-induced breakdown of the quantum anomalous Hall effect, attributed to the electric field created between opposing chiral edge states. The present work succeeds in reducing this effect, demonstrating a robustness on a par with the conventional quantum Hall effect in a semiconductor 2D electron gas. I do find this a significant and potentially useful advance, appropriate for Nature Comm.

Open Access This Peer Review File is licensed under a Creative Commons Attribution 4.0 International License, which permits use, sharing, adaptation, distribution and reproduction in any medium or format, as long as you give appropriate credit to the original author(s) and the source, provide a link to the Creative Commons license, and indicate if changes were made.
In cases where reviewers are anonymous, credit should be given to 'Anonymous Referee' and the source.
The images or other third party material in this Peer Review File are included in the article's Creative Commons license, unless indicated otherwise in a credit line to the material. If material is not included in the article's Creative Commons license and your intended use is not permitted by statutory regulation or exceeds the permitted use, you will need to obtain permission directly from the copyright holder.
To view a copy of this license, visit https://creativecommons.org/licenses/by/4.0/

We thank both Reviewers for their comments. Below we append a point-by-point response to all comments made by each Reviewer. For clarity of reading, original comments by the Reviewers are in black, and our reply to each comment is in blue.

Reviewer #1 (Remarks to the Author):

What are the noteworthy results?

The quantum anomalous Hall effect (QAHE), discovered in recent years, has attracted significant attention because of its potential applications in metrology, such as resistance standards under zero magnetic field. However, compared to the integer quantum Hall effect, practical implementation is still challenging due to a relatively small critical current, leading to skepticism regarding its feasibility.
In this paper, the authors fabricated multi-terminal Corbino devices from V-doped BiSbTe exhibiting the QAHE. By eliminating the electric field between the edge modes using electrochemical potential balancing developed by the authors, the robustness of the QAHE edge channels was investigated. The most noteworthy result is that quantum edge modes arising from the QAHE were observed even under large current bias, revealing that the edge modes are significantly more stable than previously expected. This result provides a hopeful outlook for further optimization of the QAHE in future applications.

Will the work be of significance to the field and related fields? How does it compare to the established literature? If the work is not original, please provide relevant references.

The result observed in this study is particularly significant: the quantum anomalous Hall effect (QAHE) was maintained even under current levels of several tens of microamperes, which is significantly higher than previously expected.
This demonstrates that the QAHE possesses a robustness comparable to that of the integer quantum Hall effect. Improving the quality of QAHE devices has been a global challenge, and skepticism regarding its practicality still remains.
The results findings provide a promising outlook for enhancing the stability of the QAHE in future experiments.

Does the work support the conclusions and claims, or is additional evidence needed?
Yes.
The material, device fabrication, and low-temperature transport measurements presented in this study are all of a very high standard, and the experimental results presented are sufficient to support the conclusions and claims.

Are there any flaws in the data analysis, interpretation and conclusions? Do these prohibit publication or require revision?
No.

Is the methodology sound? Does the work meet the expected standards in your field?
Yes.

We thank the Reviewer for acknowledging the importance and quality of our results. Below we address the two listed comments.

Is there enough detail provided in the methods for the work to be reproduced?

Comment1: How did the authors determine the values shown in Figs. 2B–2D? Were they calculated at a specified current, or obtained by fitting the I–V curves presented in Supplementary Information Fig. 2A?

Indeed, this is something we should have commented on from the start. The parameter values shown in Figs. 2B-2D were obtained by measuring three independent resistance configurations ($R_{A\text{-}c,A\text{-}c}$, $R_{A\text{-}C,A\text{-}C}$, and $R_{B\text{-}D,A\text{-}C}$), each measured with a small applied d.c. bias voltage of a few mV at 1.4 K (and above) and a few hundreds of µV at 30 mK. The three resistance values were then inserted into equations (3-5) in the supplement, to determine the value of each parameter (by solving the system of three equations with three unknowns). This procedure, repeated at each temperature and gate voltage generates the curves plotted in Figs. 2B-2D. We have now specified in the supplementary information that the parameters originate from calibration measurements at a low bias.

Comment2: I recommend that the authors provide information regarding the quality of the material. In the case of two-dimensional electron gases (2DEGs) such as GaAs/AlGaAs or graphene, the quality is typically characterized by carrier density and mobility. Is it possible to specify the material quality in a similar manner? Alternatively, could the authors estimate the critical current density for the breakdown of the QAHE if a usual Hall bar (rather than a Corbino geometry) were fabricated from the same material?

In the case of V-doped $(Bi,Sb)_2Te_3$, the carrier density and mobility cannot simply be obtained directly. In conventional 2DEGs, when one controls the magnetic field B (which then equals the externally applied field H, $B = \mu_0 H$), one can indeed directly extract these parameters. For our magnetic material, we cannot simply “turn off” the magnetic field, as the material is a ferromagnet and thus always has a finite magnetization M (and a finite $B = \mu_0 H + \mu_0 M$ field inside the material). Therefore, there is no way to experimentally “eliminate” the chiral edge mode, and one always has either pure chiral edge transport (QAHE) or chiral edge transport with a conducting bulk. Unfortunately, neither of the two scenarios allows for a clear way to determine the mobility and carrier density from the experiment.

With that said, if one crudely assumes single carrier transport and ignores the issues mentioned above, simply taking the longitudinal resistivity measured at zero external magnetic field and an approximate value for the slope of Hall resistance at an external field of a few tesla (well above the coercive field), in these layers one obtains a number for the carrier mobility of the order of 50 cm^2/Vs and sheet carrier density of the order of $5*10^{12}$ cm^{-2} at 4.2 K. This low apparent mobility is likely a consequence of the bulk states stemming from disorder (likely charge puddles) in these materials.

With regards to the critical current, in Ref. [21] (D. K. Patel et al. *Nat. Electron.* **7**, 1111–1116 (2024)) we have studied the QAHE breakdown in a Hall bar device fabricated from nominally identical material (epilayers grown within a few weeks from each other under nominally identical growth conditions). There we observed that the breakdown occurs above some 100 nA, which for a Hall bar of 200 µm width results in a critical current density of some $5*10^{-4}$ A/m. We have now included this information in the manuscript.

Reviewer #2 (Remarks to the Author):

The main claim of the paper is that the electrical transport in a quantum anomalous Hall insulator (QAHI) device, which employs the electrochemical potential balancing between the chiral edge modes, is “ubiquitously of quantum adiabatic nature, regardless of the temperature, position of the Fermi level in the band structure, or the electrical bias across the sample”. The

practical consequence of this approach, according to authors, could be a possibility of highly sensitive measurements in quantum metrology at temperatures above 1 K. I do not find the authors' analysis of the presented data and the conclusions drawn from it sufficiently convincing and do not recommend publishung their work in Nature Communications. Below I present my arguments:

We thank the Reviewer for their comments. Apparently, we were not clear enough in describing the scope of our study.

First, we emphasize that this is *not* a metrology paper, and any practical standard based on the QAHE operating above 1 K will still require solving material-related challenges. As we explain in more detail below, the main point of this work is a study of how robust the quantum anomalous Hall edge modes are on a *fundamental* level. We believe that our observations are a promising indication for future use in metrology, but this of course does not imply that we can directly build such standard. Below we address the Reviewer's remarks in detail.

1) Concerning the electrochemical potential balancing between the chiral edge modes, this is indeed interesting approach, which allows to avoid building up a significant electrical field between counter-propagating edge modes along the inner and outer perimeters of the Corbino device. However, it is still impossible to avoid the separation of the high and low electrochemical potentials at contacts where the current enters and exits the device. In this study, this separation at contacts A, a, C, and c is 50 mkm in the device 1 and 15 mkm in the device 2. One would expect the breakdown of QAHI state at these contacts when the current accedes a critical value. This is not fundamentally different from the situation in conventional Hall-bar devices.

Actually, this is fundamentally different from the situation in conventional Hall bar devices. In a Hall bar, at a large enough electrical bias, the inter-edge electric field opens additional current paths between the edges, for example through a network of charge puddles. Such a breakdown involving hopping is well known in this material, see for example refs. [17,25,26] in the main text. In addition to flowing along the edge modes, the electrons can then backscatter to the contacts located on the opposite edge. This acts to equilibrate the electrochemical potential throughout the sample, which decreases the Hall voltage and makes the Hall resistance deviate from h/e^2. On the other hand, in our measurement geometry, any possible breakdown at the mesa constriction (where the current enters/leaves the device) merely introduces an additional series resistance that is eliminated in a four-terminal measurement geometry. When that happens, the Hall resistance remains quantized.

With that said, it is clear from our data that no breakdown is observed in the range of tested electrical current bias (tens of microamps). The signals are stable with increasing electrical current.

2) Concerning the Landauer-Büttiker description of the mixed transport through the edge modes and the bulk channels, authors introduce a model with three parameters: \beta is a probability of scattering from an edge mode to a contra-propagating edge mode on the other perimeter of the Corbino device; R_B is an effective bulk resistance, accounting for N=1/R_B bulk channels; R_c is a contact resistance, which is assumed to be the same for all contacts. Authors treat these parameters as independent ones and obtain them from the three measured resistances in configurations shown in Supplementary Fig. 2 (three parameters from the system of three equations).
There are several problems with this model and this approach:
(i) Parameters are not independent. Both \beta and R_B related to the bulk conduction and cannot be independent from each other. This can be seen, e.g., from the equation (4) in Supplementary Materials, which essentially gives the resistance of a two-probe measurement of

a QAHI sample with a hole. If R_B is finite, \beta cannot be zero, otherwise the resistance will be quantized (h/e^2 + 2R_C), even though the sample has bulk conducting channels.

It is unclear to us why the Reviewer thinks this is a problem. Each parameter as defined describes a distinct and non-redundant transport feature of the device (β – the scattering probability from the edge mode, R_B – the resistance of the current path through the bulk, bypassing the edge mode, and R_C – the series resistance from the mesa constriction). Obviously, the parameters ultimately depend on bulk resistivity and thus will produce values which are in some way correlated with each other. This does not invalidate the parametrization.

With that said, we find it unlikely that the number of parameters in the model can be reduced to less than three, as at the very least, if all existing parameters are expressed as a function of bulk resistivity, one will have to add geometry factors, which will reintroduce fitting parameters. Based on that, and the fact that there seems to be no trivial way to express each parameter as a function of bulk resistivity (the functional dependence would have to be guessed), we argue that our present parametrization is a good compromise between simplicity (a minimum number of assumptions) and predictive power of the model (the results obtained in Figs. 3B-D are exactly as expected based on the argumentation outlined in the paper). We have previously used the same model to accurately simulate a large number of independent voltage configurations in similar devices (see Ref. [27] in the text; Fijalkowski et al. *Nat. Commun.* **12**, 5599 (2021)), which is additional proof that the model works.

(ii) It is not clear why bulk channels are connected to only opposite contacts on the inner- and outer-perimeters. As one can read from the Landauer-Büttiker conduction matrices [eqs. (1) and (2) in Supplementary Material], all N=1/R_B bulk channels, which goes into a particular contact, bring only the electrochemical potential of the opposite contact.

We agree that it is indeed beneficial to include a justification for this assumption. We have now added more discussion into the text. Below we also include a photo of the device with contact labels for clarity of reading.

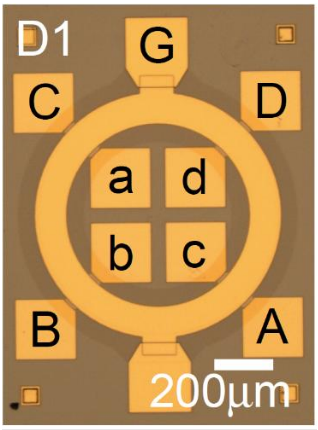

Reply Fig. 1 – Microscope image of the device with labeled contacts.

Our model presently includes a nearest neighbor path (for example between contacts A-c, labeled as R_B). The four next nearest neighbor bulk paths (from A) would include: A-B, A-b, A-d, and A-D. Note that the aspect ratio of the ring is substantial; the mesa circumference is some 28 times larger than the distance between the ring perimeters. This implies that due to geometry, each of these paths has a resistance roughly a factor of 7 larger than R_B. The factor of 7 comes from the fact that bulk path A-c has approx. 1 square, whereas bulk path A-B (as well as A-b, A-d, and A-D) has approx. 7 squares. Now looking at each bulk path one by one:

- A-B – Runs directly in parallel with the chiral edge mode. This chiral edge mode (which already is part of our model) provides an inherently very low resistance path which shorts out A and B.
- A-b – The majority of electrons that reach contact b, will follow the least resistance path given by the chiral edge mode, flowing directly to contact c. Starting from contact A, this creates a path that is in parallel to A-c, and therefore is already effectively included in the value of parameter R_B.
- A-d – Similarly to A-B, runs directly in parallel with the chiral edge mode.
- A-D – The majority of electrons that reach contact D, will follow the least resistance path given by the chiral edge mode, flowing back to contact A. This is a closed loop that has no effect on the potential profile.

We also note that the same arguments hold for paths A-C and A-a, where the geometry factor is even larger (approx. 14).

Considering all of the above, we argue that including only the nearest neighbor bulk connections (such as A-c) is a very good approximation, which does not come at the cost of making the model less realistic. We also note that this factor of 7 from geometry is an approximation. To rigorously include such paths in the model, one would have to add geometry factors as separate fitting parameters. This in our view would unnecessarily complicate the model, without a clear benefit.

(iii) Contact resistance R_C seems to be one of the most important parameters in the proposed approach. It allows by simply adding I_0*R_C to V_B and subtracting I_0*R_C from V_D to obtain effective voltages, which are independent from the gate voltage, the temperature or the bias current and their difference gives the quantized Hall resistance (as shown in the Fig. 3). Judging from the gate and temperature dependencies of R_C, it does not look as a real contact resistance, but rather as some effective parameter without clear physical meaning. R_C can be even negative at gate voltage of -2.7V as one can see in Supplementary Fig. 2 B. On the other hand, real contact resistances of this device can be easily measured in the three-probe configuration, at least at 30 mK, when QAHI state is not broken.

From the Reviewer's remark, we realize we did not explain the meaning of the parameter R_C in a sufficiently understandable way. It was also probably unwise from our side to call it a "contact/lead resistance", which indeed may cause confusion. We have now branded it as a "series resistance" (with label changed to R_S in the manuscript, below we will refer to it with this new label) and added more description into the main text to clarify what it is.

The physical meaning of the parameter R_S is quite simple. It is the resistance of the mesa constriction between each lead and the Corbino ring. This "series resistance" R_S results from both bulk and edge channel transport through this constriction. Note that the constriction is covered by the top gate in our devices, which makes R_S dependent on the applied gate voltage. Crucially, the gate voltage dependence of R_S qualitatively follows the measured longitudinal resistivity of the sample at each temperature, which itself is a direct measure of the combined resistance from bulk and edge states (See longitudinal resistance in Supplementary Information Fig. 4B). In our view this robustly justifies our interpretation of what R_S is.

As to the slightly negative value of R_S right at the side of the QAHE plateau at 30 mK, this is most likely an artifact resulting from bulk resistivity not being uniform enough along the ring when the bulk states are barely at the mobility edge. Obviously, our simple modelling assumes perfectly symmetric device and homogeneous material, and spatial variations will introduce uncertainties. Importantly, this does not occur at higher temperatures (which is the focus of this study) where inhomogeneities are effectively screened by the conducting bulk and transport is

uniform. This is clearly reflected by the quality of the data and the model analysis in Fig. 3B-D (the results are exactly as expected based on the arguments made in the text, which implies that the model gives a good description of transport in that regime). We have added a comment on this in the supplementary information.

3) In general, the proposed approach is as follows: from resistances measured in several configurations on a Corbino device, certain parameters (whatever their physical meaning) are extracted and then all transport components, including the contribution of edge modes, are reconstructed.
The accuracy of the reconstruction depends on the model and sample details, making this approach fundamentally problematic for quantum metrology, which the authors invoke as a motivation for their study.

As stated earlier, in this work we aim to explore fundamental properties of topological edge modes. It is not intended as a blueprint to build a quantum standard, as the material challenge (making the bulk insulating above 1 K) remains. This is beyond the scope of this paper.

More specifically, what was not known prior to this work, is whether the dissipationless edge modes in a magnetic topological insulator can carry large currents at high temperatures (tens of microamps at 4.2 K), which is one of the necessary requirements to efficiently use a cryogenic current comparator and thus, build a stable primary standard in the future. In fact, prior studies, reporting on apparent fragility of the QAHE indicated that such current levels may be fundamentally out of reach. In our manuscript, we show that the dissipationless nature of transport in the QAHE edge modes is similarly robust as that in the quantum Hall effect. This has not been demonstrated before and is certainly a promising sign for future practical use in metrology.

The claim of the quantum adiabatic nature of the transport in presented device is also not justified, in my opinion. My understanding is that the adiabaticity in QHE (QAHI) systems implies dissipationless 1D edge transport which is responsible for a precise quantization of the Hall resistance. The gate voltage, the temperature or the current can break this state, and I do not see how the proposed reconstruction can change this fact. Authors need to make it clear what they mean by the adiabaticity, at least make a section in Supplementary Material.

The Reviewer's remark is incorrect. We realize we may not have provided a clear enough description of what "quantum adiabatic transport" is. This is important enough to include in the main text, rather than in the supplement, and now we have substantially elaborated on that.

Quantum transport effects always have a certain characteristic energy scale, such that usually when the environment (e.g. thermal energy), external stimuli (e.g. electrical bias), or any other possible source puts too much energy into the system, exceeding this characteristic scale, quantum effect diminishes. As an example, one can take a quantum point contact (at zero external magnetic field). To measure quantized electrical conductance, one must ensure that *both* temperature T and electrical bias V are low enough, such that the energy put into the system (be it $k_B T$ or eV) is smaller than the energy separation from quantum confinement.

One of the fascinating aspects of the quantum Hall effect is that the transport behavior is fundamentally different from the one described above. In the quantum Hall effect, the relevant energy scale is given by the energy separation between the Landau levels, which usually is of the order of a few meV. Indeed, when the thermal energy $k_B T$ is large enough to thermally activate the carriers to the higher Landau level, the quantization of electrical conductance breaks down. However, the same does not happen when the energy comes from electrical bias. One can apply a voltage of the order of 1 V to the edge modes, which corresponds to an energy scale orders of

magnitude larger than the Landau level separation, and yet one can still observe perfectly quantized transport without any spurious dissipation. Such large voltages are crucial for the stable operation of cryogenic current comparators. Indeed, this is routinely done in metrology, where, for example, measurements in graphene show precise quantization of Hall resistance (to within 1 × 10^{-9}) for bias currents up to 0.5 mA, which corresponds to a Hall voltage of some 6 V (see Ref. [31] in the text; Ribeiro-Palau et al., *Nat. Nanotech.* **10**, 965–971 (2015)). This is quantum adiabatic transport: A 1D quantized dissipationless transport regime characterized by a full suppression of scattering (see Ref. [29] in the text; Beenakker and van Houten, *Solid State Physics* **44**, 1-228 (1991)), which ultimately is protected at the topological level. The key point is that the experiments on the integer quantum Hall effect show that this protection appears to work for a seemingly arbitrarily large electrochemical potential of the edge mode, which clearly differentiates it from conventional ballistic quantized transport (such as that of a quantum point contact).

This *intrinsic* robustness of the edge transport must be distinguished from the device limitations. Obviously in real life experiments there are some practical limits to what can be measured, due to, for example, heating effects at the hot spots or a dielectric breakdown through the bulk states at large enough bias. Our focus here is entirely on *intrinsic* robustness of the 1D transport along the edge of a quantum anomalous Hall insulator, which prior to this work remained an open problem.

In previous studies of the QAHE samples, the experimental detection of this kind of large-bias dissipationless transport regime was entirely impossible due to device limitations (in a conventional Hall bar geometry, a relatively small electrical bias already causes scattering between the edges through the bulk). In the present paper, electrochemical potential balancing is used to mitigate this problem, allowing us to test the dissipationless nature of the edge modes up to a substantially larger electrical bias, detecting similarly robust transport as that of the integer quantum Hall effect. In our opinion, this is a major observation, especially given how fragile the QAHE is currently believed to be. In this context, the Reviewer's statement "The gate voltage, the temperature or the current can break this state" is incorrect. Our experiments show that dissipationless nature of the transport along the edge of a quantum anomalous Hall insulator is prevalent, and seemingly neither of the three parameters mentioned by the Referee can "break" that (obviously, as long as the sample remains ferromagnetic). This is the ultimate demonstration of how robust the topological protection is in a topological insulator.

We also note that the equipotential along the edge (dissipationless transport along the ring) is an experimental observable (see Supplementary Information Fig. 5B, where the *measured* potentials at contacts B and C are always equal; top part of each panel). Therefore, the statement of presence of dissipationless transport does not rely on any "reconstruction" or model considerations.

4) What is the point of comparing the bias voltage with the thermal energy scale k_B*T/e?

The key feature of quantum adiabatic transport is that almost arbitrarily large electrical bias energy can be supplied to the edge mode without a breakdown of its quantized dissipationless properties. The comparison to the relevant energy scales in the experiment ($k_B T$ is one of them) merely serves to underline this. We realize we may have put too much emphasis on that single specific energy scale. We have now changed the wording here and there.

We believe that we have been able to address all the Reviewers' concerns.

Below we provide a point-by-point reply. For ease of reading, the original comments by the Reviewers are in black and our reply is in blue.

Reviewer #1 (Remarks to the Author):

The authors have responded to my comments thoroughly. The manuscript is of very high scientific quality. I can now support publication.

We thank the Reviewer for their time and effort in reviewing this manuscript.

Reviewer #2 (Remarks to the Author):

The authors have made significant revisions to the manuscript and addressed most of my comments, but some questions remain. In their response, authors emphasize that the paper is not aimed at building a metrological standard, but rather focus on the robustness of the QAH edge states, for which authors added the explanation of the idea of quantum adiabaticity in several parts of the main text and SM.

Nevertheless, the metrology aspect remains the primary motivation for paper, and the question remains as to how accurate the quantization of the Hall resistance is at different temperatures, gate voltages, and applied bias currents.

This paper is not about metrology, it is about quantum adiabatic transport. Quantum adiabatic transport is a basic feature of the QHE, but prior to this work, it was not known whether this is also true for the QAHE (the Hall bar measurements that the Referee mentions towards the end of their review do not prove anything with regards to that, we comment on that later on). Our first observation of quantum adiabatic transport in a quantum anomalous Hall insulator is indeed an important prerequisite towards establishing a standard based on the QAHE, but building such a standard is not the topic of this manuscript

The specific question about the precision of quantization that can be reached in metrology under such conditions requires achieving a perfectly insulating bulk at 4.2 K (only then can one directly measure Hall resistance quantization using a CCC). This is a considerable materials-related challenge which will likely take years to address. The Referee is moving the goal posts. This is far beyond the scope of this study and is not necessary to support any claim that we make.

Authors claim that “any possible breakdown at the mesa constriction (where the current enters/leaves the device) merely introduces an additional series resistance that is eliminated in a four-terminal measurement geometry. When that happens, the Hall resistance remains quantized”. I doubt this statement. It is based on the reconstruction of the edge and the bulk contributions to the conductance from three experimentally measured resistances $R_{A-c,A-c}$, $R_{A-C,A-C}$, and $R_{B-D,A-C}$ as shown in Fig.3. The estimation of the edge contribution close to e^2/h is not a surprise, simply because it was implemented into the Landauer-Büttiker model.

The Referee apparently does not understand the basics of a four-terminal measurement. Our statement is not based on any Landauer-Büttiker analysis or reconstruction; it is far more basic than that. In their first report, the Reviewer pointed to a possible mechanism that effectively increases contact resistance, and nothing else (adding a series resistance due to scattering inside of the mesa constriction where the current enters/leaves the device). Our four-terminal

detection method eliminates the influence of any such series resistance. Therefore, the Hall signal remains unchanged by it (this mechanism would only affect a two-terminal measurement). This is something that has been known since 1861, when Lord Kelvin invented the four-terminal measurement technique to avoid series resistance.

Having said that, we again emphasize that this point is completely irrelevant to anything we discuss in the manuscript. We do not observe anything in our data that would suggest that a contact resistance induced breakdown is occuring.

This reconstruction also plays a central role in the consideration of the transport adiabaticity.

No, it does not. Again, as we've explained before, we have *directly measured* an equipotential along the ring, which is direct evidence for dissipationless transport along the edge. This is an *observable* and does not rely on any reconstruction or modelling. Given this observation, we use the model to further analyze the signals and rigorously test them against the expected Landauer-Büttiker transport, but the conclusion of our paper is based on a direct measurement.

In particular, authors added the following statement in the revised version of the manuscript: the applied "voltage is some 1700 times larger then kBT/e at 4.2 K and orders of magnitude larger than any known relevant energy scale associated with the quantum anomalous Hall state. This is a clear evidence for quantum adiabatic transport [29]; dissipationless transport with a full suppression of any inter-edge channel scattering, holding for an almost arbitrarily large electrochemical potential of the edge."
The concept of adiabatic transport in the field of QHE, which was reviewed in the seminal paper by Beenakker and van Houten and cited in the paper as Ref. [29], is related to the suppression of the inter-edge channel scattering, which can destroy the quantization of the transport response (quantized Hall resistance and zero longitudinal one). The edge channel in QAHI is obviously differs from the edge channels in QHE (with possible exception of the state with a filling factor one). Nevertheless, the scattering between counter-propagating edge modes on the inner- and outer-perimeters of the Corbino device can destroy the adiabatic nature of the edge modes. In fact, the reconstruction of transport parameters (Fig.2) shows finite values of the parameter \beta (except for the lowest temperature 30 mK and the Fermi level position within the magnetic gap). This is a direct indication of a finite scattering probability between these two edge modes, pointing on the possible loss of the adiabatic edge transport.

First, it is still up for debate how the edge modes of the QAHE differ from those of the QHE. In both cases, they are characterized by topological quantum numbers that differ from those of bulk states. The Reviewer's definitive statement that they "obviously" differ is not evidence based.

The second point the Referee makes, about the finite value of β, is not relevant for the transport regime we focus on (where the potential at both edges is equal). A finite value of β is merely equivalent to stating that the bulk is conducting, but for any scattering to occur, a voltage drop is needed to drive it. Therefore, an electron flowing in the edge mode can short circuit to the opposite edge through the bulk *only when there is a potential difference between the edges to drive the current*. The actual number of scattered electrons will be proportional to *both* β and this potential difference. We have now added an additional comment in the manuscript to make this point even clearer than before.

The absence of a voltage across the bulk is essential for the experiment we discuss in the paper. We show that when we use electrochemical potential balancing, which brings the potential of both adjacent edges to the same value, dissipationless transport is *directly measured* (by observing the equipotential along the edge), which must imply that scattering is suppressed.

Any scattering would create a voltage drop along the ring, as energy would be lost, which is *not* what we measure. This shows that the transport *is* adiabatic under these conditions.

Another point that requires further consideration/clarification in the paper concerns the assumption that the applied bias voltage multiplied by the elementary charge represents the relevant energy scale for adiabatic quantum transport. Even in the quantum Hall effect, such an energy scale cannot be directly applied for comparison with the Landau level separation energy. Why should it be relevant for the quantum anomalous Hall effect?
In the quantum point contact example given by authors in in their response, temperature and applied bias voltage are also not the only relevant parameters. Arguably the spatial dependence of the confinement potential plays a more important role. So, the relevance of the energy scale in the nature of the adiabatic transport should be properly justified.

Of course the electrical bias is relevant. It is unclear to us why the Reviewer thinks that some energy scales in the system can simply be ignored. Quantum transport is all about comparing the energy scale protecting given quantum phenomena to the energy scales imposed by the experiment/environment. Electrical bias *is directly applied to the electron system* and thus gives the energy to the electrons, and that energy scale is given by $e * V$. It is a fact that under such conditions, the electrons in the edge mode gain a large excess energy from the bias (the correspondingly large measured voltage proves that).

The Referee dismisses relevance of this energy, as if the reason why the quantization is so robust against the bias current was obvious. It most certainly is not, even in the context of conventional QHE. The remarkable robustness of the QHE edge modes (and as we conclude from our results, also the QAHE edge modes) implies that there must be some other aspect protecting the state against large energy from the electrical bias. The fact that edge states have a different topology from the bulk states (and are characterized by a different quantum number for each Landau level) is likely of relevance here. To eliminate the quantized transport, one either would have to excite the carriers to a higher topological level (if allowed by quantum number) or lose the topological quantum number by heating. While significant heating does likely occur at the hot spots, it may not be possible to alter the global topological properties of the system with the electrical current alone. Critically, all that is still speculative, and on a fundamental level, not well understood in any system, including the traditional QHE. The Referee is again moving the goal posts. A comprehensive explanation of the "relevance of the energy scale in the nature of the adiabatic transport" is far beyond the scope of this paper, nor is it needed to support our claims. Transport in the QHE is generally accepted to be of quantum adiabatic nature, and in this manuscript, we merely show that, contrary to common belief that the state is extremely fragile, the QAHE edge modes have similar robust properties, *i.e.* transport is also adiabatic.

With regards to the quantum point contact, it is debatable what parameter is more relevant. Obviously, the shape of the potential plays a role, but if the temperature or electrical bias is large enough, the quantization will break down no matter the details of the potential profile. However, we do not see how this is relevant for anything discussed in the paper.

Despite the questions raised, the overall conclusion of the paper—that the edge modes in the ferromagnetically ordered QAHI state are quite robust—is plausible. After all, the typical temperature dependences of the resistivities in usual QAHI Hall-bar samples show a rapid increase of the anomalous Hall signal in R_{yx} and a rapid decrease of R_{xx}, indicating an increasing contribution to the conductance from the edge channel which is at least comparable with the bulk contribution even at temperatures close to the Curie temperature. And this behavior does not depend much on the current, which can be easily tens of microamps.

The Referee is again missing the point of our paper, which is showing the quantum adiabatic transport. None of the prior experiments conducted on Hall bars have been capable of demonstrating that. The key difference between our approach and that using Hall bars, is that none of the studies on Hall bars have shown *dissipationless* transport at such high temperatures and currents. Transport in a Hall bar under such conditions is always dissipative, making it impossible to conclude anything about dissipationless (or adiabatic) properties of the edge modes under such conditions. There is a major difference between speculating and providing empirical evidence for such adiabatic transport.

Overall, this work is a fairly valuable contribution to the QAHE research, however, in my opinion, the reported results are not important enough to justify publication in Nature Communication.

We obviously disagree with the Reviewer's opinion about the impact of this paper and its suitability for Nature Communications. We report on the occurrence of quantum adiabatic transport in a material system for which this was previously thought impossible. By any means, this observation is groundbreaking, which justifies dissemination in a high-profile journal.

For clarity of reading, the Referees' comments are in black, and our reply is in blue.

Reviewer #2 (Remarks to the Author):

The authors' response didn't change my opinion of the manuscript, and their sarcasm —" The Referee apparently does not understand the basics of a four-terminal measurement ... known since 1861, when Lord Kelvin invented the four-terminal measurement technique "— isn't helping either. While I don't support the publication of this article in Nature Communications, I think it's only fair that readers form their own opinions about the scientific merit of this work.

No offence was intended, but identifying that the Referee was arguing from a point which has long been known to be wrong is a scientifically appropriate response.

As to the last sentence, we fully agree. Fortunately, given Nature Communications transparent peer review policy, should the paper be accepted at Nature Communications, the readers will have access to the manuscript and the review process, so they will be well placed to form their own opinion on the scientific merit of the work.

Reviewer #3 (Remarks to the Author):

This manuscript wirtten by K. M. Fijalkowski et al. reports the observation of quantum adiabatic transport in a quantum anomalous Hall insulator using a multi-terminal Corbino geometry combined with an electrochemical potential balancing scheme. The authors demonstrate that dissipationless edge transport persists under electrical bias up to ~600 mV at 4.2 K, substantially exceeding previously reported bias limits in QAHE systems. The work is technically impressive and of potential importance for quantum metrology and the understanding of topological edge transport. The manuscript is well written, and the experimental methodology appears sound. I recommend its publication in Nature Communication, provided the authors address the following comments.

We are pleased to receive this positive assessment of the quality and impact of our work, and thank the Reviewer for raising several interesting and important questions, which we address as follows:

1.At a maximum bias voltage of 600 mV and a current of around 17.5uA, the total injected power into the mesoscopic device exceeds 10uW. While the edge modes themselves are dissipationless, the current flowing through the bulk and the series mesa constrictions will inevitably produce Joule heating. Does this localized heating affect the bulk resistivity or the inter-edge scattering probability? A brief discussion on the thermal dissipation dynamics would solidify the robustness claim.

While some heating is certainly present in any such dissipative channel, here we do not observe any measurable effect of such, as the current-voltage characteristics are linear throughout the entire tested bias range (we discuss this more, and show data, in our reply to the Reviewer's second comment below). Therefore, at least for the experiment at hand, this localized heating appears to have no measurable effect on the bulk resistivity, as if it did, it would manifest as non-linearity in the *I*-*V* characteristic of the mesa constriction R_S (which depends on the bulk resistivity). Furthermore, given the measured equipotential along the device (in the balanced bias scheme), heating does not affect the inter-edge scattering, which remains suppressed at large bias. We have added a discussion of these points near the end of page 4 in the text.

2.The conclusion of quantum adiabatic transport appears to rely on the agreement between the measured contact potentials and the Landauer-Büttiker model after correcting for the series resistance Rs. Could the authors comment on how sensitive is the conclusion to uncertainties in the extracted Rs values? Does Rs remain Ohmic up to 17.5 uA? Non-linearities in the contact and constriction resistance at high bias could undermine the accuracy of the V\pm I0Rs correction, It would be helpful if the authors could provide an I-V curve of the constriction itself or demonstrate the constancy of Rs at higher bias current.

R_S is linear up to 34 uA to within our experimental resolution of about 1%, as we now show in Supplementary Information Fig. 6 (and explain in a bit more detail below). This implies a roughly similar uncertainty on the conclusion, which is to say that if there was 1% dissipation in the edge state, we would resolve that.

To obtain the *I-V* characteristic of the mesa constriction, a "three-terminal" measurement [(V_A-V_B) as a function of I_0] is analyzed, which is the closest one can come to measuring this characteristic in a device with the layout we employ. The path between the nodes *A* and *A'* is the mesa constriction resistance R_S (together with any contact resistance), and contact *B* probes the potential at node *A'* due to dissipationless nature of edge transport. Therefore, the voltage drop V_A-V_B gives a measure of the voltage drop over R_S (and contacts). Indeed, such a three-terminal setting is commonly used to estimate the contact resistance in quantum Hall devices. The resulting *I-V* characteristic (Reply Fig. 1B; Reply Fig. 1 reproduces the Supplementary Information Fig. 6) is linear to within approx. 1%, tested up to I_0 of some 34 μA at a temperature of 4.2 K. For completeness, in Reply Fig. 1C we also show the corresponding *I-V* characteristic of the total voltage applied to the sample V_A (measured at the same time), which is linear to within approx. 0.1% in the same current range. The overall response is ohmic to within the resolution of our experiment.

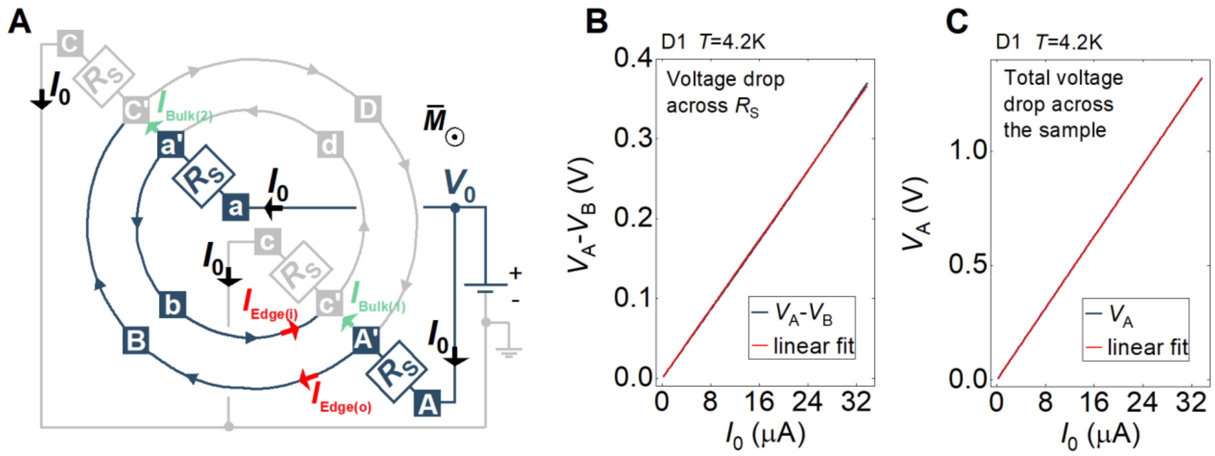

Reply Fig. 1 *A) Schematic of the balanced electrical bias setup with labeled contacts. B) Bias current dependence (I_0) of the voltage drop V_A-V_B, which gives a measure of the voltage drop over the mesa constriction R_S. Blue line is the data collected from device D1 at a temperature of 4.2 K and gate voltage of 4 V. Red line is a linear fit. C) The same for a total voltage applied across the sample (V_A).*

Having said that, we stress that the conclusion about the occurrence of quantum adiabatic transport can already be made based solely on the fact that we *measure* an equipotential along the ring (see Supplementary Information Fig. 5), which demonstrates the absence of backscattering, all prior to any Landauer-Büttiker analysis.

3.Although the electrochemical potential balancing scheme suppresses the net inter-edge current, a finite bulk conductance RB-1 is still present in the model. Could the authors further

clarify to what extent residual bulk transport may influence the extracted edge potentials, particularly at elevated temperatures or large bias?

There is no influence of the bulk conductance, other than the fact that it affects R_S. A great advantage of this approach is that under the voltage-balanced bias scheme, the current entering the device (I_0) splits into two separate paths, one flowing along the ring via the dissipationless chiral edge mode, and the other flowing through the bulk, as represented in Fig. 3A in the main text. Based on our observations, this remains true regardless of the value of bulk conductance. By changing various experimental parameters (such as temperature and gate voltage), we tune the bulk conductance in the sample (which can be observed as changes in the value of $1/R_B$) by several orders of magnitude, and the resulting electrical potentials match those expected for a quantized dissipationless transport. We have added a further comment to that to the main text.

4.The authors state that dissipationless transport holds "at least up to an applied bias voltage of ~600 mV" (or ~700 mV for I0 = 17.5 uA). Did the authors directly observe a breakdown of quantization beyond this voltage, or is this limit set by more trivial experimental constraints?

This is indeed an important point to clarify, and now we do so. We have not observed any breakdown in this experiment, so presumably one could go to even larger bias. Indeed, the reasons we did not go higher than ~1 V is due to an instrumental limitation of the precision voltage amplifiers that we use to measure the potentials.

5.The comparison with conventional quantum Hall systems is interesting. Could the authors provide a more quantitative comparison in terms of current density, breakdown thresholds, etc, to better establish the claimed equivalence in metrological performance?

Given that electro-chemical potential balancing has yet to be properly implemented for a conventional quantum Hall experiment (this is something we are currently working towards, together with our colleagues from metrology institutes), a quantitative comparison is challenging. In conventional quantum Hall metrology devices (using a large Hall bar geometry) the breakdown occurs when the voltage difference between the edges exceeds some critical value, which eventually enables backscattering through the bulk. Our measurement geometry eliminates this voltage drop, leaving a finite electric field only near the hot spots at the contacts, which makes it a fundamentally distinct setting. Indeed, a direct comparison of both effects under the balanced bias scheme would be very interesting. We have now added a comment in the manuscript.

So, for now only measurements on a standard Hall bar of both anomalous and conventional effects can be compared quantitatively. As we already discuss in the manuscript, the quantum anomalous Hall effect is substantially less robust than the quantum Hall effect, looking at any metric (because bulk conductance can be very easily activated). Indeed, this is the exact reason why moving to the voltage-balanced scheme is so advantageous, as it mitigates some of these material-related drawbacks.

Therefore, presently only a qualitative comparison is possible. All we can conclude from our study thus far is that one can inject (at least) some 20 μA into the quantum anomalous Hall edge mode while maintaining dissipationless transport, which is already a value similar to the current routinely used for the quantum Hall effect in metrology. This shows that on a fundamental level, the transport in the quantum anomalous Hall edges appears similarly robust to that of the quantum Hall edges. This was not known to be true prior to the present study.
